# Dual Benefits of Endophytic *Bacillus velezensis* Amzn015: Growth Promotion and Root Rot Control in *Atractylodes macrocephala*

**DOI:** 10.3390/microorganisms13102300

**Published:** 2025-10-03

**Authors:** Na Zhu, Jiongyi Wu, Sen Fan, Qingling Meng, Shijie Dai, Mingjiang Mao, Weichun Zhao, Xiaofeng Yuan

**Affiliations:** School of Life Sciences, Zhejiang Chinese Medical University, Hangzhou 310000, China; zhuna85@163.com (N.Z.); wjy591011306@163.com (J.W.); fansen2001@163.com (S.F.); 15192866713@163.com (Q.M.); dsj2513140@163.com (S.D.); zcmummj2021@163.com (M.M.); weichunzhao@zcmu.edu.cn (W.Z.)

**Keywords:** *Bacillus velezensis* Amzn015, *Atractylodes macrocephala*, root rot, systemic resistance, biological control, continuous cropping obstacles

## Abstract

*Atractylodes macrocephala* Koidz. (*A. macrocephala*), a medicinal plant extensively used in traditional Chinese medicine, is greatly susceptible to root rot under continuous monoculture, leading to serious yield and quality losses. To develop a sustainable control strategy, we isolated the endophytic bacterium *Bacillus velezensis* (*B. velezensis*) Amzn015 from healthy *A. macrocephala* plants and assessed its biocontrol efficacy and underlying mechanisms. In vitro assays showed that Amzn015 significantly inhibited *Fusarium oxysporum* and other phytopathogenic fungi by disrupting hyphal morphology and reducing spore viability. Pot experiments confirmed its effectiveness in reducing disease incidence and promoting plant growth. Mechanistically, Amzn015 induced reactive oxygen species accumulation and upregulated key defense responsive genes involved in salicylic acid, jasmonic acid/ethylene, and phenylpropanoid signaling pathways. The findings imply that Amzn015 synchronously activates systemic acquired resistance and induced systemic resistance in *A. macrocephala*. This dual activation contributes to enhanced immunity and plant vigor under pathogen challenge. Our findings offer fresh perspectives on the biocontrol potential of endophytic *B. velezensis* Amzn015 and support its application as an eco-friendly agent for managing root rot in medicinal crops.

## 1. Introduction

*Atractylodes macrocephala* Koidz. (*A. macrocephala*) is a perennial herb of the genus *Atractylodes* in the family Asteraceae, and its dried rhizome serves as a fundamental medicinal ingredient in traditional Chinese medicine. It is widely used for its core therapeutic functions, including tonifying the spleen, replenishing qi, dispelling dampness, and promoting diuresis [1]. The total cultivation area of *A. macrocephala* in China exceeds 200,000 mu (one mu is approximately equal to 666.67 square meters), with major production regions including Anhui, Jiangxi, Henan, Hebei, Hubei, and Zhejiang. Among these, *A. macrocephala* from Zhejiang is especially renowned for its geo-authenticity and superior quality [2]. However, long-term continuous cropping has led to severe growth disorders in *A. macrocephala*, such as plant dwarfism, leaf chlorosis, and fibrous root degradation. These issues have significantly limited its yield and quality, posing a major bottleneck to the sustainable utilization of this valuable medicinal resource and hindering the development of its cultivation industry.

One of the most critical challenges associated with continuous cropping of *A. macrocephala* is root rot, which has become the primary disease restricting its sustainable cultivation. Typical symptoms include longitudinal cracks, damage to the root epidermis with cambial tissue necrosis, light-brown lesions and black spots, and the emergence of white, flocculent mycelia on the root surface [3]. In severely affected regions, root rot can cause production losses of up to 60%. Studies have shown that Fusarium spp. dominate the rhizosphere and endophytic fungal communities of diseased plants, posing a serious threat to *A. macrocephala* health [4]. Current disease management strategies primarily rely on chemical pesticides, which carry the risks of environmental residues and the development of pathogen resistance [5]. Al Raish et al. pointed out that the practical application of plant-growth-promoting microorganisms (PGPMs) in agriculture is constrained by multiple factors, including the instability of field performance, limited survival and colonization under natural environmental conditions, and obstacles related to regulatory approval and application methods [6]. In contrast, green and efficient biological control approaches remain underdeveloped and are urgently needed.

In this context, endophytes—symbiotic microorganisms residing within plant tissues—have attracted increasing attention for their ability to promote host health through diverse mechanisms. These include direct antagonism against phytopathogens [7], stimulation of plant growth [8], and increase in stress tolerance [9]. Among them, *Bacillus velezensis* (*B. velezensis*) has emerged as a particularly promising biocontrol agent due to its capacity to produce a wide array of antimicrobial compounds, such as lipopeptide antibiotics and cell wall-degrading enzymes [10], Several strains have demonstrated notable efficacy in agricultural applications: *B. velezensis* 8-2 effectively reduces the disease index of cherry leaf curl [11], *B. velezensis* SX13 promotes cucumber growth [12], and *B. velezensis* F9 enhances cucumber seedling vigor via indole-3-acetic acid (IAA) secretion and nitrogen fixation [13]. These findings suggest that *B. velezensis* strains possess diverse functional traits conducive to sustainable crop protection and productivity.

Despite these advancements, the application of *B. velezensis* in the biological control of *A. macrocephala* root rot remains insufficiently explored, particularly in the context of alleviating continuous cropping-related obstacles. It remains unclear whether specific strains such as Amzn015 can effectively mitigate *Fusarium* infection by synergistically activating induced systemic resistance (ISR). Addressing this knowledge gap is critical for developing green disease management strategies and ensuring the safety and quality of medicinal plant resources.

In this study, the endophytic strain *B. velezensis* Amzn015 was isolated from healthy *A. macrocephala* plants in Zhejiang Province. Its antagonistic activity against *Fusarium oxysporum* (*F*. *oxysporum*), along with its growth-promoting and resistance-inducing capabilities, was systematically investigated. We further explored the underlying mechanisms by which Amzn015 enhances host defense, focusing on its ability to regulate reactive oxygen species (ROS) metabolism and activate systemic resistance pathways. This work aims to provide both theoretical insights and practical strategies for the eco-friendly control of *A. macrocephala* root rot and to support the sustainable cultivation of this important medicinal crop under continuous cropping conditions.

## 2. Materials and Methods

### 2.1. Sample Preparation and Microbial Isolation

Rhizome samples of *A. macrocephala* were collected from a cultivation base in Panan, Zhejiang Province. After washing with tap water and sterile distilled water, the samples were surface-sterilized by sequential immersion in 3% NaClO for 1 min and 75% ethanol for 3 min, followed by three thorough rinses with sterile water. To assess the effectiveness of surface sterilization, 100 μL of the final rinse water was collected, spread onto lysogeny broth (LB) agar plates (Hangzhou Microbial Reagent, Hangzhou, China), and incubated to detect microbial growth. The sterilized rhizomes were then homogenized using an automatic rapid sample grinder (Shanghai Jingxin, Shanghai, China), and the resulting homogenate was subjected to a 10-fold serial dilution (10^−1^ to 10^−5^). Aliquots (100 μL) of each dilution were spread onto LB agar plates and incubated at 37 °C for 24 h to isolate rhizosphere-associated microorganisms. Individual colonies were purified, assigned serial numbers beginning with Amzn001, and stored in 80% (*v*/*v*) glycerol at −80 °C for future use.

### 2.2. Preliminary Screening of Antagonistic Bacteria Against F. oxysporum

A strain of *F. oxysporum* was obtained from Chuanglian Biotechnology Institute (Chaoyang District, Beijing, China). The optimal antagonistic bacterial strains were screened using a dual-culture plate assay [14]. *F. oxysporum* was cultured on potato dextrose agar (PDA) at 28 °C. An 8 mm diameter mycelial plug was taken from the edge of an actively growing colony using a sterile cork borer and placed at the center of a fresh PDA plate. Ten microliters of fermentation broth from each bacterial isolate was then spotted in a cross (“+”) pattern 2 cm away from the fungal plug, while an equivalent volume of LB medium served as the negative control. Plates were incubated at 28 °C for up to 9 days, or until the control fungus had fully overgrown the plate. After incubation, the colony radius of *F. oxysporum* was measured on both the control plate (Rck) and the treatment plate (Rd). We calculated the inhibition rate (V) with the following expression: V = [(Rck−Rd)/(Rck−Rm)] × 100 [15], where Rck is the colony radius on the control plate, Rd is the colony radius on the treatment plate, and Rm is the radius of the initial fungal disc (Rm = 4 mm), all measured in millimeters.

### 2.3. Verification of Broad-Apectrum Antagonistic Activity

To verify the inhibitory spectrum of the optimal antagonistic endophytic bacterium against other pathogenic fungi associated with continuous cropping obstacles in *A. macrocephala*, a dual-culture plate assay was conducted as described above. The antagonistic activity was tested against three fungal pathogens previously isolated from diseased *A. macrocephala* plants in our laboratory: *Fusarium foetens*, *Epicoccum catenisporum*, and *Fusarium arcuatisporum*. The molecular identification of these pathogens is shown in Supplementary Figure S1. All fungal strains used in this assay were preserved in our laboratory culture collection.

### 2.4. Morphological and Molecular Identification of Amzn015

Gram staining was conducted as described [16]. The strain was incubated on LB agar at 37 °C for 18–24 h, and its cell morphology was then examined with a light microscope (CX40, Sunny Group, Zhejiang Province, China). Genomic DNA was extracted using a commercial kit (Tiangen, Beijing, China), and the 16S rRNA gene was amplified using primers reported previously [17]. The amplified products were sequenced on an Illumina HiSeq 2500 platform (Illumina, San Diego, CA, USA) at Zhejiang Easy-Decode Biotechnology Co., Ltd. The resulting sequences were analyzed using BLAST (National Center for Biotechnology Information) and GraphPad Prism software (version 9.5.0; GraphPad Software, San Diego, CA, USA) for homology comparison. Subsequently, a phylogenetic tree was constructed with the neighbor-joining algorithm in MEGA version 11 (www.megasoftware.net; accessed on 17 December 2024). Whole-genome sequencing of Amzn015 was conducted on the PacBio platform (BGI, Wuhan, China). High-quality DNA was extracted from three independent bacterial samples (DNA integrity number ≥7.0; concentration ≥20 ng/μL), followed by library preparation and sequencing. Reads were aligned to a reference genome using BWA (Burrows-Wheeler Aligner, version v0.7.17), and variant calling was performed with GATK (Genome Analysis Toolkit, version v4.4.0.0). The raw sequencing data have been deposited in the NCBI database under accession number PRJNA1294532.

### 2.5. Assessment of Amzn015’s Plant Growth Capabilities

To investigate the plant-growth-promoting mechanisms of strain Amzn015, its nitrogen fixation ability, siderophore production, and phosphate solubilization capacity were assessed following the established methods [18]. IAA production was quantified using the Salkowski colorimetric assay as described in the cited protocol [19].

### 2.6. Effects of Amzn015 on Spore Germination, Viability, and Hyphal Morphology of F. oxysporum

#### 2.6.1. Effect on Spore Germination

An equal volume of Amzn015 bacterial suspension (in logarithmic phase) and *F. oxysporum* spore suspension (10^7^ CFU/mL) was mixed at a 1:1 ratio and co-incubated in a 96-well plate for 12 h. The control group received an equal volume of LB medium instead of bacterial suspension. Spore germination was monitored by light microscopy, and both total and germinated spores were quantified using a hemocytometer (Biosharp, Anhui, China). Germination rate was calculated accordingly, and each treatment was conducted in triplicate.

#### 2.6.2. Assessment of Spore Viability by Evans Blue Staining

Amzn015 and *F. oxysporum* suspensions (both at 10^8^ CFU/mL) were added to potato dextrose broth (PDB; Hangzhou Microbial Reagent, Hangzhou, China) to a final concentration of 3% (*v*/*v*) and co-cultured for 3 days. Mycelia were removed using sterile gauze (Solarbio, Beijing, China), and the resulting spore suspension was stained with 0.05% Evans Blue and incubated at ambient temperature for 10 min [20]. Spores were then observed under a light microscope, and Viable spores were quantified using a hemocytometer, and these counts were used to calculate the survival rate. The control group received an equal volume of sterile PDB.

#### 2.6.3. Scanning Electron Microscopy (SEM) Observation of Hyphae

To observe hyphal morphology, Amzn015 and *F. oxysporum* were co-cultured using the dual-culture plate assay described above. Hyphae from the interaction zone were collected and fixed in 1.5% glutaraldehyde at room temperature (approximately 20–25 °C) for 1–2 h. The samples were washed three times with distilled water and then dehydrated using 90% tert-butanol. After dehydration, samples were placed in a vacuum freeze dryer (VFD-21S, SHINKKU VD, Fujisawa, Japan) for 2 h and observed under a scanning electron microscope (SU8010, Hitachi, Japan). Micrographs were recorded as described [21].

### 2.7. Assessment of the Effects of Amzn015 on the Germination and Seedling Performance of A. macrocephala

Healthy seeds of *A. macrocephala* were selected and surface-sterilized by immersion in 3% NaClO for 1 min and 75% ethanol for 3 min, followed by five rinses with sterile water. Seeds were blotted dry on sterile filter paper. Strain Amzn015 was cultured in LB broth at 37 °C with shaking until the logarithmic phase, after which bacterial cells were harvested by centrifugation and resuspended in sterile PBS to a final concentration of 10^8^ CFU/mL. Sterile filter paper was placed in Petri dishes, and 5 mL of bacterial suspension was added; the control group received 5 mL of PBS. Fifteen sterilized seeds were evenly placed in each dish. Plates were incubated in a growth chamber (Boxun, Shanghai, China) at 25 ± 1 °C with a 16 h light/8 h dark photoperiod and maintained at 70% relative humidity. Water lost to evaporation was replenished daily. On day 12, we determined germination percentage (GP%) using the equation: GP (%) = (number of germinated seeds/total seeds) × 100 [22]. Germinated seeds were transplanted into a sterilized soil mix consisting of vermiculite, perlite, and sterile soil in a 1:1:3 ratio. At the two-leaf stage, entire seedlings were carefully removed. Roots were rinsed to remove attached soil and submerged in water to fully straighten. Root length and shoot height were measured using a ruler. Shoot height was defined as the distance from the soil surface to the tip of the tallest leaf. Whole seedlings were submerged in distilled water within sealed bags for 24 h to attain full turgor. After removing excess moisture with filter paper, fresh weight was measured. The seedlings were then cut into small pieces, placed in glass Petri dishes, and dried in a forced-air oven at 115 ± 10 °C for 30 min, followed by further drying at 65 °C for 6 h until a constant weight was reached. Dry weight was recorded using an electronic balance (Shimadzu ATX224R, Kyoto, Japan).

### 2.8. Assessment of Disease Index in A. macrocephala Following F. oxysporum Infection with or Without Amzn015 Induction

Healthy six-week-old *A. macrocephala* seedlings at the two-leaf stage were selected for pot experiments. *F. oxysporum* was cultured on PDA plates at 28 °C for 7 days. Mycelia from the colony edge were transferred to PDB and incubated at 28 °C with shaking at 180 rpm (THZ-98A, Shanghai, China) for 5 days. The culture was filtered through 12 layers of sterile gauze to remove mycelia, and the resulting spore suspension was adjusted to 10^8^ CFU/mL in sterile PBS using a hemocytometer. Strain Amzn015 was initially cultured on LB agar plates at 37 °C for 12 h and a single colony was inoculated into LB broth and incubated at 37 °C with shaking at 180 rpm for 48 h. The bacterial suspension was then adjusted to 10^8^ CFU/mL with sterile PBS. Two-leaf-stage seedlings were transplanted into sterile plastic pots containing a soil mix of vermiculite: perlite: sterile soil (1:1:3). Roots were rinsed with sterile water and plants were divided into four groups, each transplanted into individual pots. Seven days before fungal inoculation, the Amzn015 treatment group was root-drenched with 10 mL of Amzn015 suspension (10^8^ CFU/mL). The co-treatment group (Amzn015 + *F. oxysporum*) and the infection control group (*F. oxysporum*) were inoculated by root-drenching with 10 mL of *F. oxysporum* spore suspension (10^8^ CFU/mL). Once obvious disease symptoms appeared on the leaves, Amzn015 suspension (10 mL) was applied to the co-treatment group by root drenching. The PBS control group (CK) received an equal volume of sterile PBS. Whole seedlings from all treatments were harvested at 0, 6, 12, 24, and 48 h post-inoculation, wrapped in aluminum foil, snap-frozen in liquid nitrogen, and stored at −80 °C for further analysis. Leaf disease severity was evaluated on day 65 using a 0–4 scale based on the proportion of symptomatic (yellowing or wilting) leaves as follows: 0 = no symptoms; 1 = 1–33% of leaves affected; 2 = 34–66% of leaves affected; 3 = 67–100% of leaves affected; 4 = whole plant dead. This scoring system followed the method described by Tang et al. [23]. The disease index and relative control efficacy were calculated using the following formulas: Disease index (DI, %) = [∑ (number of plants in each category × corresponding score)/(total number of plants × highest score)] × 100. Control efficacy (%) = [(disease index in control−disease index in treatment)/disease index in control] × 100 [24].

### 2.9. Determination of Defense Enzyme Activities in A. macrocephala Induced by Amzn015

To verify the resistance induced by Amzn015 in *A. macrocephala*, defense enzyme activities were measured in infected seedlings [23]. Seedlings from both control and treatment groups were collected at 0, 6, 12, 24, and 48 h post-inoculation. Roots, stems, and leaves were harvested and homogenized on ice using a tissue grinder, with a tissue mass (g) to extraction buffer volume (mL) ratio of 1: 10. The homogenates were centrifuged at 8000 rpm for 10 min at 4 °C, and the supernatants were transferred to 1.5 mL centrifuge tubes for enzyme assays. Peroxidase (POD), superoxide dismutase (SOD), catalase (CAT), superoxide anion radical (O_2_^−^), and malondialdehyde (MDA) levels were determined using commercial assay kits (Solarbio, Beijing, China) in accordance with the manufacturer’s instructions. Reaction reagents were added to both control and measurement tubes, mixed thoroughly, and absorbance (OD) values were measured using a microplate reader (Eppendorf D30, Berlin, Germany). Enzyme activities were calculated based on the respective formulas provided in the assay protocols.

### 2.10. RNA Extraction, cDNA Synthesis, and qPCR Analysis

Total RNA was isolated from plant material flash-frozen in liquid nitrogen using the FastPure Plant Total RNA Isolation Kit (Vazyme, Nanjing, China). RNA concentration and purity were measured with a NanoDrop One™ spectrophotometer (Thermo Fisher Scientific, MA, USA). One microgram of RNA was then reverse-transcribed into cDNA using the HiScript IV All—in—One Ultra RT SuperMix for qPCR (Vazyme). Quantitative real-time PCR (qRT–PCR) was carried out on a QuantStudio™ 3 system (Thermo Fisher Scientific, MA, USA) with SYBR Green I dye (Thermo Fisher) to quantify gene expression levels. The reaction system was set up based on established protocols described in previous studies [25]. The *Amactin* gene (actin) was used as the internal reference. Target genes analyzed included *AmPR1* (pathogenesis-related protein 1), *AmPAL* (phenylalanine ammonia-lyase), *AmERF1* (ethylene response factor 1), *AmICS* (isochorismate synthase), *AmPR4* (pathogenesis-related protein 4), *AmETR2* (ethylene receptor 2), *AmPR2* (pathogenesis-related protein 2), *AmNPR1* (nonexpressor of pathogenesis-related genes 1), *AmJAR1* (jasmonate-resistant 1), *AmC4H* (cinnamate-4-hydroxylase), *AmCAD* (cinnamyl alcohol dehydrogenase), *Am4CL* (4-coumarate:CoA ligase), *AmWRKY26*, and *AmWRKY22* (WRKY transcription factors). Primer sequences are listed in Supplementary Table S1. Amplification specificity was confirmed via melt-curve analysis and verified by agarose gel electrophoresis. Relative expression levels were calculated using the 2^−ΔΔCT^ method [26] based on three biological replicates.

### 2.11. Statistical Analysis

One-way analysis of variance (ANOVA) was used to compare differences among groups. Graphs were generated using GraphPad Prism version 9.5.0 (GraphPad Software, San Diego, CA, USA). The results are presented as mean ± standard deviation (SD).

## 3. Results

### 3.1. Isolation of Endophytic Bacteria from A. macrocephala and Screening for Antagonistic Activity

**Figure 1:**
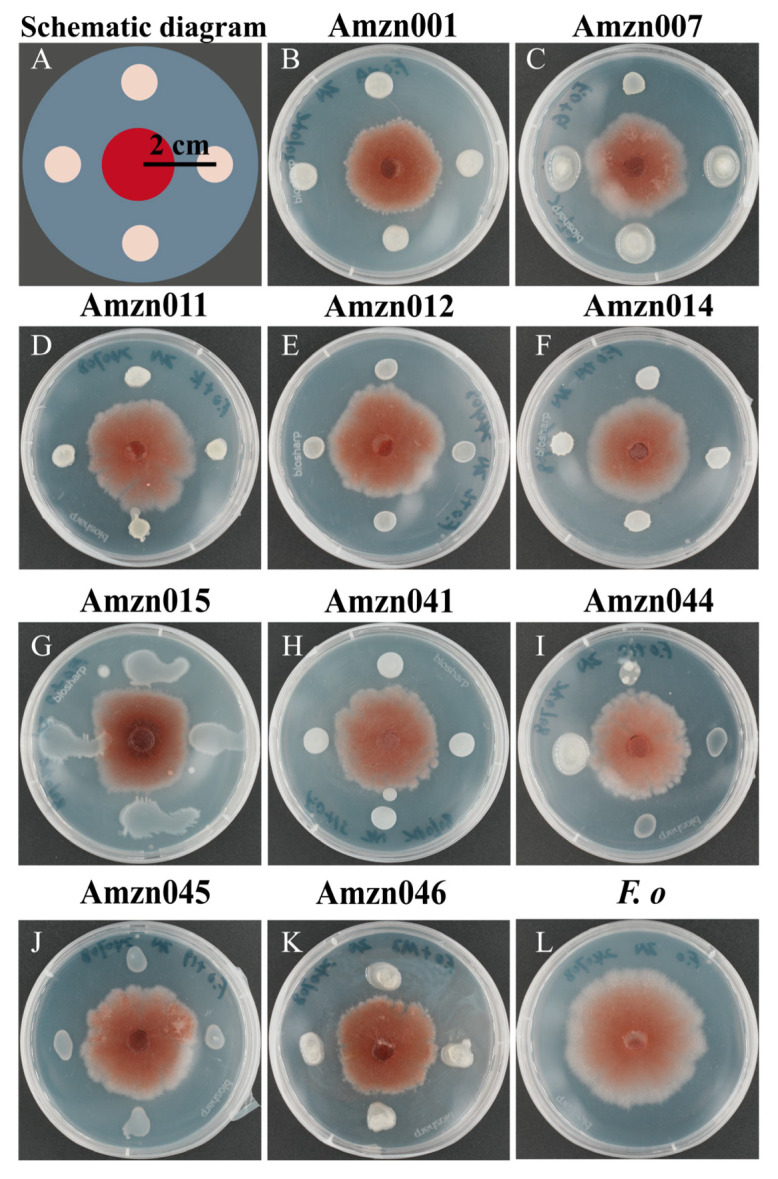
Dual culture assays of ten endophytic bacterial strains against *F*. *oxysporum* on PDA plates. (**A**) Schematic representation of the dual culture setup; (**B**–**K**) inhibition of *F. oxysporum* by strains Amzn001, Amzn007, Amzn011, Amzn012, Amzn014, Amzn015, Amzn041, Amzn044, Amzn045, and Amzn046, respectively; (**L**) *F. oxysporum* grown alone as a control. (*n* = 3).

A total of 46 endophytic bacterial strains (designated Amzn001–Amzn046) were isolated from the rhizomes of healthy *A. macrocephala* plants collected in Pan’an, Zhejiang Province. In vitro antagonism assays revealed that 10 of these isolates exhibited inhibitory activity against the root rot pathogen *F. oxysporum* (Figure 1). Among them, strain Amzn015 demonstrated the most pronounced antagonistic effect, with an average inhibition zone of 2.33 ± 0.08 cm (Supplementary Table S2).

**Figure 2:**
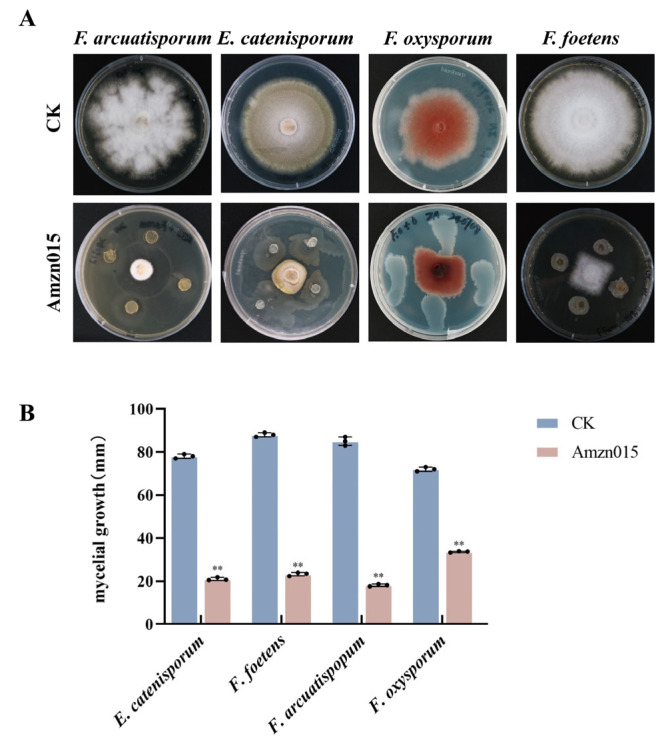
Antagonistic activity of the biocontrol strain Amzn015 against four pathogenic fungi isolated from diseased *A. macrocephala*. (**A**) Dual-culture confrontation assays were performed on PDA plates. CK represents the control group, in which the pathogen was inoculated alone without any treatment. The four tested pathogens include *F. oxysporum*, *F. foetens*, *E. catenisporum*, and *F. arcuatisporum*. Clear inhibition zones indicate that Amzn015 exhibits strong antagonistic activity against all four pathogens. (**B**) Quantitative analysis of fungal diameter. (*n* = 3). ** *p* < 0.01.

In addition, Amzn015 exhibited strong antifungal activity against other common pathogens associated with *A. macrocephala*, including *E. catenisporum*, *F. arcuatisporum*, and *F. foetens* (Figure 2). Based on these results, Amzn015 was selected for further characterization in this study.

### 3.2. Amzn015 was Identified as Bacillus velezensis

**Figure 3:**
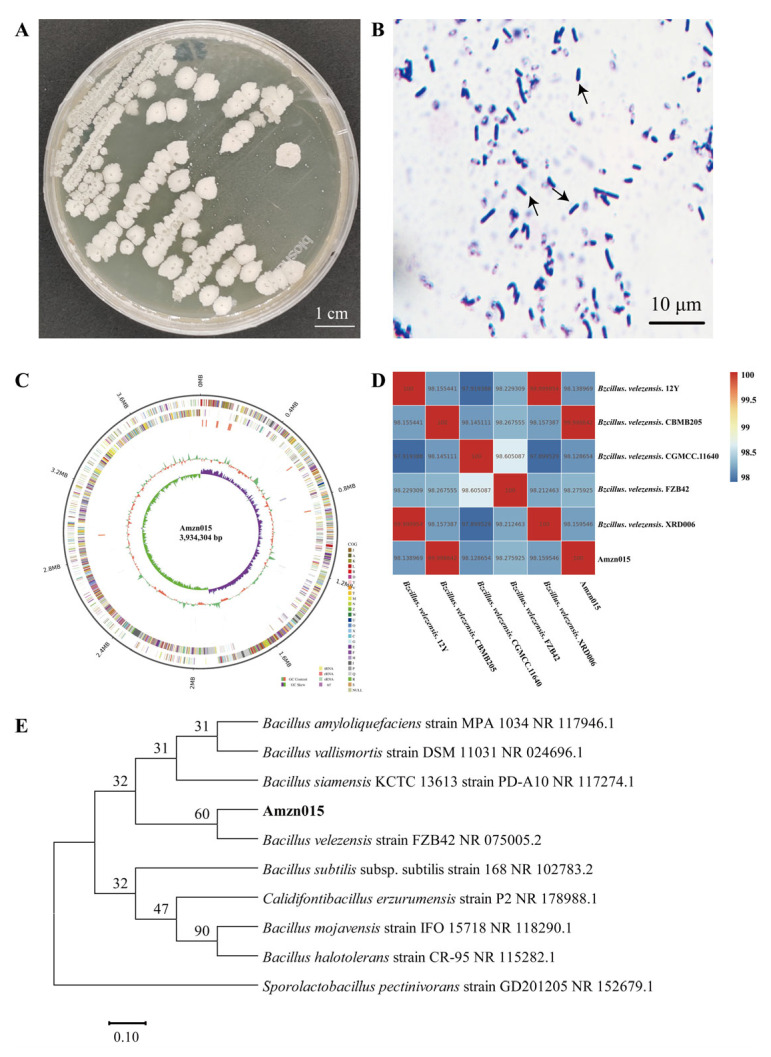
Phenotypic, genomic, and phylogenetic characterization of strain Amzn015. (**A**) Colony morphology of Amzn015 on LB agar plates. (**B**) Gram staining observed under oil immersion microscopy; black arrows indicate Gram-positive bacteria. (**C**) Circular chromosome map of Amzn015 generated via the CGView server. (**D**) Average nucleotide identity (ANI) heatmap comparing Amzn015 with related bacterial genomes; both axes represent genome names, and values indicate ANI percentages. (**E**) Neighbor-joining phylogenetic tree based on 16s rRNA gene sequences, showing the evolutionary relationship between Amzn015 and closely related Bacillus strains. (*n* = 3).

Strain Amzn015 formed milky white, circular colonies with diameters of 5–8 mm, irregular edges, and a dry, wrinkled surface (Figure 3A). Gram staining revealed that the cells were Gram-positive and rod-shaped (Figure 3B). The 16s rRNA gene sequence (PV972215) and whole-genome data (PRJNA1294532) for Amzn015 were obtained and deposited in the NCBI database (https://www.ncbi.nlm.nih.gov/genbank/, accessed on 17 July 2025). The circular genome map of Amzn015 is shown in Figure 3C. Phylogenetic analysis based on the 16s rRNA gene indicated that Amzn015 clustered within the *Bacillus velezensis* clade (Figure 3E). In addition, average nucleotide identity (ANI) analysis confirmed a high degree of genomic similarity (98–99%) between Amzn015 and reference strains of *B. velezensis* deposited in the NCBI database (Figure 3D). Taken together, the morphological characteristics and molecular evidence supported the identification of Amzn015 as *Bacillus velezensis*.

### 3.3. Microscopic Evidence Reveals the Multitarget Antagonistic Mechanism of Amzn015

**Figure 4:**
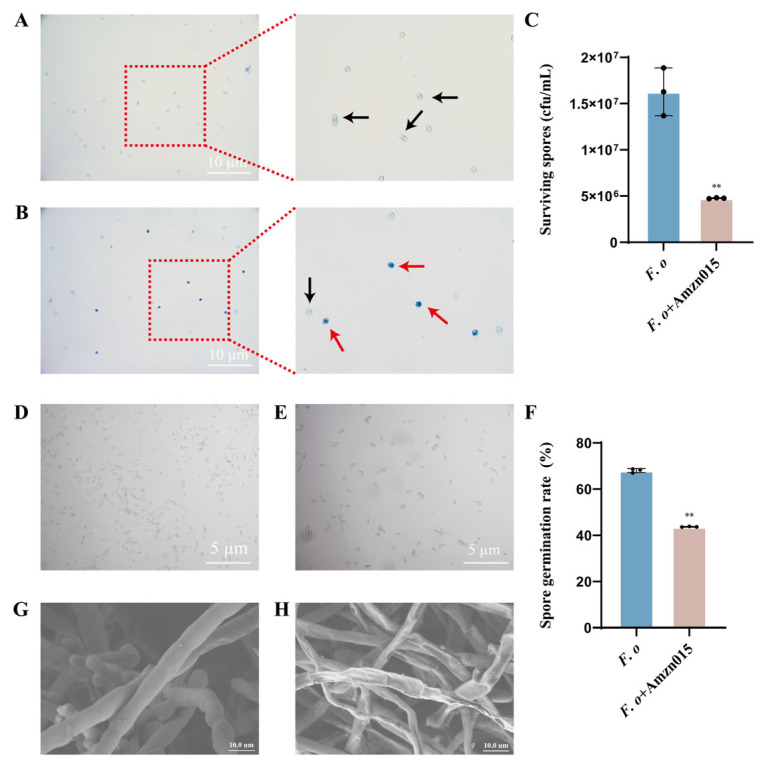
Microscopic observation of spore viability and hyphal morphology of *F. oxysporum* after treatment with Amzn015. (**A**,**B**) Evans blue staining of *F. oxysporum* spores under different treatments. The black arrow indicates viable spores, and the red arrow indicates non-viable spores. (*F. o: F. oxysporum* inoculation alone; *F. o* + Amzn015: combined treatment with Amzn015 and *F. oxysporum*.) (**C**) Quantitative analysis of *F. oxysporum* spore density. (**D**) Germination morphology of *F. oxysporum* spores. (**E**) Impaired germination morphology of *F. oxysporum* spores after Amzn015 treatment. (**F**) Statistical analysis of spore germination rates under different treatments. (**G**,**H**) SEM image of *F. oxysporum* hyphae under different treatment. (*n* = 3). ** *p* < 0.01.

Evans Blue staining indicated that spores in the *F. o* group remained viable, showing minimal staining (Figure 4A), whereas spores treated with Amzn015 exhibited a markedly increased proportion of blue-stained cells (Figure 4B), suggesting a loss of membrane integrity and cell viability. Quantitative analysis showed that the spore survival rate in the *F. o* + Amzn015 group decreased by 70.8% compared to the control (Figure 4C). In addition, light microscopy revealed that Amzn015 significantly inhibited spore germination, compared with the *F. o* group, the germination rate of the *F. o* + Amzn015 group decreased by 35.8% (Figure 4D–F). Scanning electron microscopy (SEM) further confirmed the direct damaging effects of Amzn015 on *F. oxysporum* hyphae. Hyphae in the treated group exhibited severe shrinkage and deformation (Figure 4H), while those in the *F. o* group retained a smooth and turgid morphology (Figure 4G). These results collectively demonstrate that Amzn015 exerts a multitarget antagonistic effect on the pathogen by disrupting spore viability, inhibiting germination, and damaging hyphal structures.

### 3.4. Amzn015 Enhances Growth and Disease Resistance in A. macrocephala

**Figure 5:**
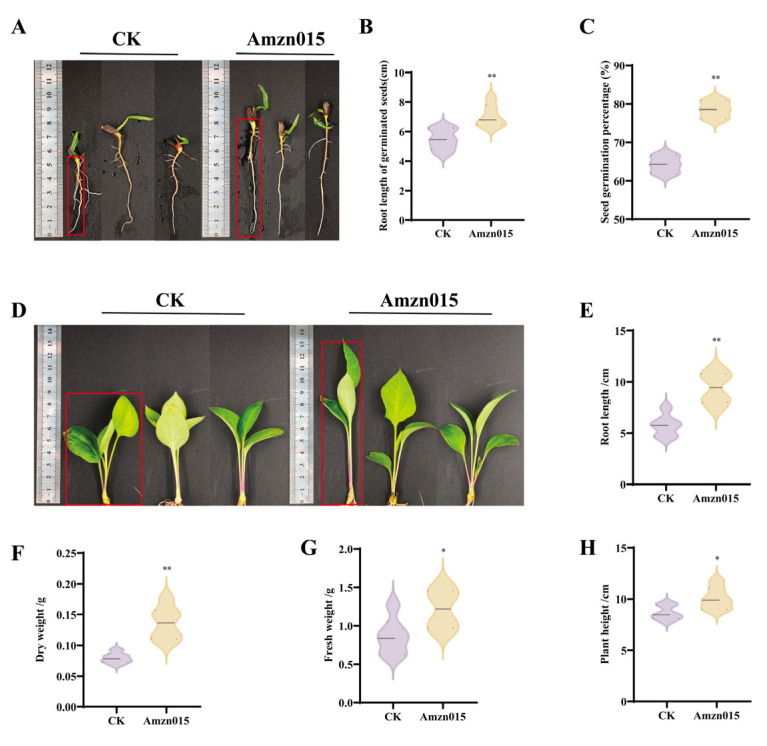
Effects of strain Amzn015 on seed germination and seedling growth of *A. macrocephala*. (**A**) Representative image of seed germination under different treatments. The red box highlights the differences in root length among *A. macrocephala* seeds. (CK: distilled water control group; Amzn015: Amzn015 inoculation alone.) (**B**,**C**) Root length and germination rate of seeds. (**D**) Representative images of *A. macrocephala* seedling height under different treatments. The red box highlights the differences in shoot height among *A. macrocephala* seedlings. Quantitative analysis of root length (**E**), dry weight (**F**), fresh weight (**G**), and plant height (**H**) of seedlings after 20 days of growth. (*n* = 6). * *p* < 0.05, ** *p* < 0.01.

Compared to the CK group, treatment with Amzn015 increased the seed germination rate of *A. macrocephala* by 22.2% (Figure 5C). Significant improvements were also observed in seedling growth parameters: shoot height increased by 16.6% (Figure 5E), root length by 63.5% (Figure 5F), dry weight by 72.0% (Figure 5G), and fresh weight by 40.9% (Figure 5H), indicating a robust plant-growth-promoting effect.

**Figure 6:**
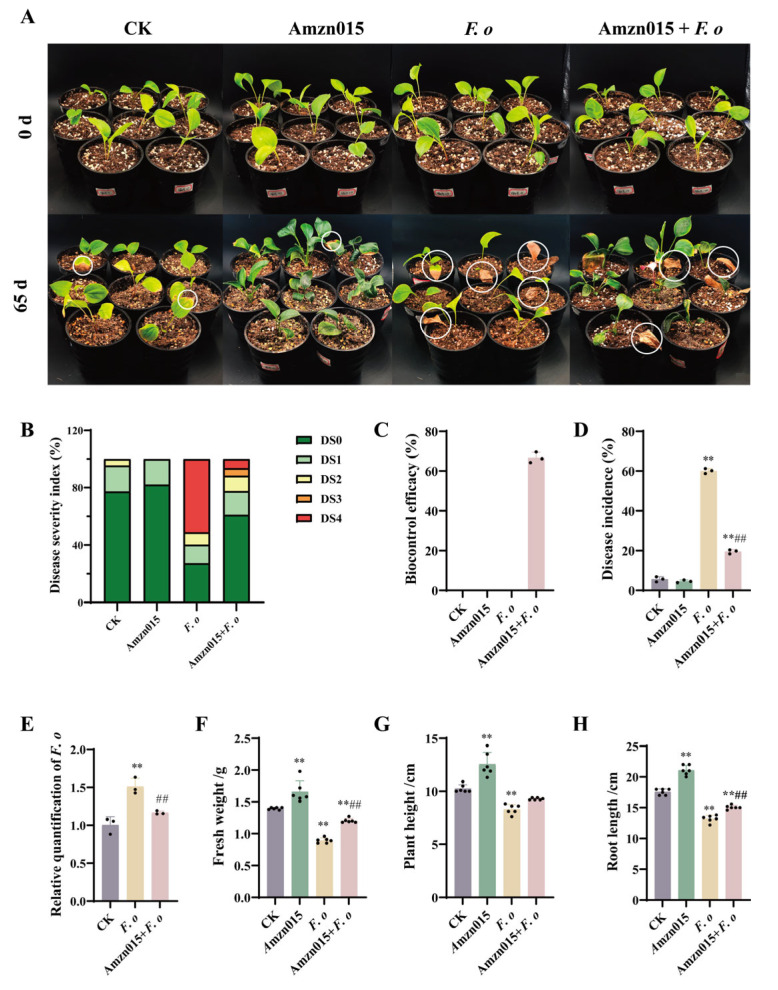
Pot experiment evaluating the biocontrol efficacy of strain Amzn015 against root rot of *A. macrocephala.* (**A**) Representative images of seedling phenotypes under different treatments. (CK: PBS control; *F. o: F. oxysporum* inoculation alone; Amzn015: Amzn015 treatment alone; *F. o* + Amzn015: combined treatment with Amzn015 and *F. oxysporum*.) (**B**–**H**) Disease progression and plant growth parameters were assessed 65 days post-inoculation, including: (**B**) disease index, (**C**) relative control efficacy, (**D**) disease incidence, (**E**) *F. oxysporum* biomass, (**F**) seedling fresh weight, (**G**) shoot height, and (**H**) root length. (*n* = 6). ** *p* < 0.01. ^##^ *p* < 0.01.

There was no significant difference in disease symptoms between the CK and the Amzn015 group (Figure 6A), indicating that Amzn015 itself was non-pathogenic to the host. The disease incidence of the *F. o* group reached 58.7%, while *F. o* + Amzn015 reduced the index to 19.9% (Figure 6D), resulting in a relative control efficacy of 66.2% (Figure 6C). In addition, the biomass of *F. oxysporum* in the *F. o* + Amzn015 group decreased by 33.0% (Figure 6E). Under pathogen stress, the *F. o* + Amzn015 group significantly mitigated growth suppression compared to the *F. o* group, with fresh weight increasing by 34.9% (Figure 6F), shoot height by 11.8% (Figure 6G), and root length by 14.1% (Figure 6H). These results suggest that Amzn015 not only suppresses root rot but also promotes plant recovery and growth under biotic stress.

### 3.5. Amzn015 Enhances Disease Resistance in A. macrocephala by Dynamically Regulating ROS Metabolism

**Figure 7:**
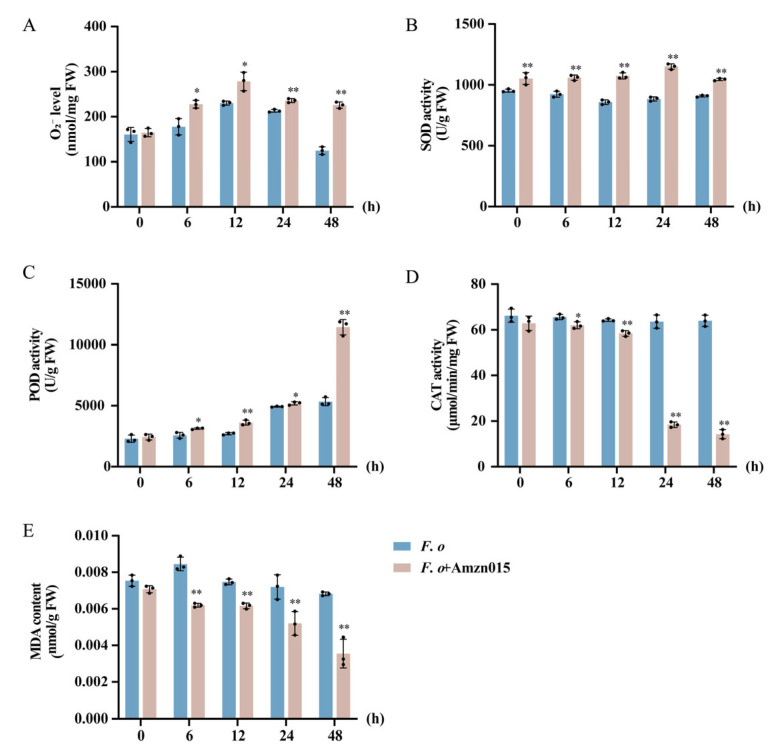
Effects of Amzn015 on antioxidant enzyme activities in *A. macrocephala* seedlings. (**A**) Superoxide anion radical (O_2_^−^) production. (**B**) Superoxide dismutase (SOD) activity. (**C**) Peroxidase (POD) activity. (**D**) Catalase (CAT) activity. (**E**) Malondialdehyde (MDA) content. (*F. o: F. oxysporum* inoculation alone; *F. o* + Amzn015: combined treatment with Amzn015 and *F. oxysporum*.) (*n* = 3). * *p* < 0.05, ** *p* < 0.01.

A dynamic interplay exists in plants between ROS and the antioxidant defense system. In Figure 7A, seedlings of *A. macrocephala* in the *F. o* + Amzn015 group displayed a short-lived increase in O_2_^−^ levels, along with progressive rises in SOD (Figure 7B) and POD activities (Figure 7C), while CAT activity gradually decreased (Figure 7D). This suggests that Amzn015 rebalances cellular oxidation, bolstering ROS-mediated defense during *F. oxysporum* infection. Meanwhile, lowered MDA levels point to reduced lipid peroxidation (Figure 7E), indicating mitigation of oxidative damage despite elevated ROS production. Overall, Amzn015 appears to modulate host oxidative stress by amplifying ROS generation and specific antioxidant enzyme activities, strengthening the plant’s defense while limiting cellular damage.

### 3.6. Amzn015 Activates the Disease Resistance Signaling Network in A. macrocephala

**Figure 8:**
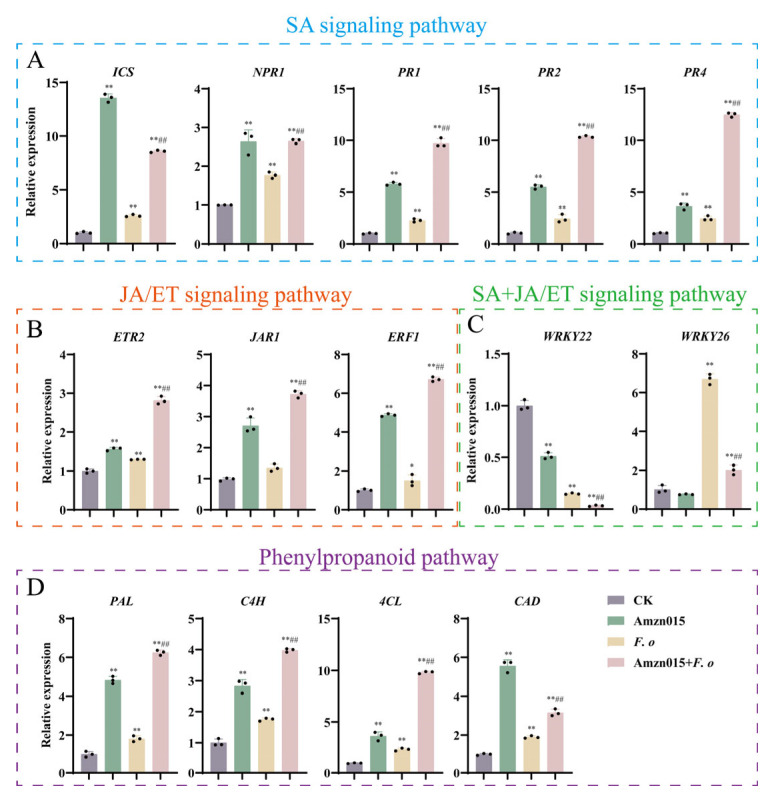
Relative expression levels of key defense-related genes in the SA, JA/ET, and phenylpropanoid pathways in *A. macrocephala*. (CK: PBS control; *F. o: F. oxysporum* inoculation alone; Amzn015: Amzn015 treatment alone; *F. o* + Amzn015: combined treatment with Amzn015 and *F. oxysporum*.) Expression changes for genes in three defense pathways: SA pathway (*ICS*, *NPR1*, *PR1*, *PR2*, *PR4*) (**A**), JA/ET pathway (*JAR1*, *ERF1*, *ETR2*) (**B**), WRKY22, WRKY26 in both SA and JA/ET pathway (**C**), and phenylpropanoid biosynthesis pathway (*PAL*, *C4H*, *4CL*, *CAD*) (**D**). (*n* = 3). * *p* < 0.05, ** *p* < 0.01 compared with the CK group. ^##^ *p* < 0.01 compared with the *F. o* group.

Plants fine-tune these pathways through complex signaling interplay to optimize defense strategies. Figure 8 illustrates that Amzn015 markedly enhances defense-gene expression across all three pathways. In the SA mediated SAR pathway, core genes (*ICS*, *NPR1*, *PR1*, *PR2*, *PR4*) showed a pronounced peak at 24 h under the *F. o* + Amzn015 group, far exceeding levels in the *F. o* group. Similarly, in the JA/ET dependent ISR pathway, *JAR1* and *ERF1* were steadily upregulated from 0 to 24 h, and *ETR2* expression surged most strongly during the *F. o* + Amzn015 group. Finally, key phenylpropanoid biosynthesis genes (*PAL*, *C4H*, *4CL*, *CAD*) were rapidly induced at 24 h in the Amzn015 group and *F. o* + Amzn015 group, suggesting an early boost in antimicrobial compound production and cell wall reinforcement. Notably, our data show that in the *F.o* + Amzn015 group, *WRKY22* and *WRKY26* were actually downregulated compared to the *F.o* group alone, indicating that Amzn015 exerts a suppressive effect on these WRKY transcription factors during infection.

## 4. Discussion

*A. macrocephala*, one of the “Zhebawei” (eight renowned medicinal herbs of Zhejiang), is widely used for its spleen-strengthening and qi-tonifying properties. However, its large-scale cultivation is increasingly threatened by continuous cropping obstacles, particularly root rot caused by fungal pathogens. Among these, *F. oxysporum* has been identified as a major causal agent. In agricultural biocontrol, various beneficial microbes have demonstrated promising results. For instance, Bacillus subtilis and certain Trichoderma spp. have been commercialized and successfully applied in field control of cotton Verticillium wilt [27]. Nevertheless, the effectiveness of these exogenously applied microbes in open-field environments is often limited by environmental fluctuations, changes in the rhizosphere microenvironment, and host immune responses. These factors can reduce colonization efficiency and lead to inconsistent biocontrol performance, thereby restricting their broader agricultural application [28]. Additionally, many endophytes possess dual functions in promoting plant growth and enhancing stress tolerance [29], and their ecological safety profile is generally superior to that of exogenous microbial agents. Therefore, the use of endophytic microorganisms represents a highly promising strategy for overcoming continuous cropping obstacles in *A. macrocephala* cultivation. Their intrinsic compatibility with the host, long-term functional stability, and multifaceted benefits position them as valuable tools in the sustainable and modernized production of medicinal plants.

We isolated Amzn015, a broad-spectrum antifungal endophyte from *A. macrocephala*, and identified it via morphology, physiological tests, and whole-genome sequencing as *Bacillus velezensis*. In dual-culture assays, Amzn015 exhibited strong inhibitory activity against key fungal pathogens, including *F. oxysporum*, *E. catenisporum*, *F. arcuatisporum*, and *F. foetens*. This study suggests that Amzn015 likely inhibits hyphal growth by inactivating *F. oxysporum* spores and disrupting spore germination. For comparison, on PDA medium, *B. velezensis* strain B.BV10 achieved approximately 47% mycelial growth inhibition (MGI) against *F. oxysporum* [30]; whereas our strain Amzn015 showed a stronger suppression rate of 56.9 %. In greenhouse trials, root drenching with Amzn015 reduced root rot severity by 38.8% compared to the *F. o* group and demonstrated therapeutic effects in already-infected plants, and had a growth recovery effect on already infected plants. This is the first documented use of *B. velezensis* Amzn015 against root rot in *A. macrocephala*, offering experimental support for its potential in large-scale medicinal plant disease management.

In addition to disease suppression, Amzn015 demonstrated significant plant growth-promotion (PGP) effects in *A. macrocephala* [31]. Twenty days of treatment led to notable increases in dry and fresh biomass, shoot height, and root length—indicators of enhanced vigor and stress resilience [32,33]. Remarkably, Amzn015 also restored growth in infected seedlings to levels comparable to healthy controls, aligning with reports that biocontrol strains can aid recovery in pathogen-challenged plants [34]. This dual PGP and biocontrol potential echoes observations in other Bacillus species—such as *B. velezensis* CE100, which suppresses fungal pathogens via chitinase/β–1, 3–glucanase production while synthesizing IAA to stimulate root growth and improve yield in strawberries. Likewise, *B. amyloliquefaciens* HF–01 reduced postharvest rot in citrus without sacrificing yield or quality [35]. Thus, *B. velezensis* Amzn015 not only controls disease but also enhances growth and recovery—offering a compelling case for its integrated use in medicinal plant cultivation, aligning with its multifunctional profiles documented in related *Bacillus* strains.

Building on the observed mitigation of root rot symptoms, our data further indicate that Amzn015 orchestrates a finely tuned oxidative defense akin to a plant-pathogen biochemical “arms race” in *A. macrocephala*. Consistent with previous findings that biocontrol inducers modulate activities of key antioxidant enzymes POD, SOD, and CAT during pathogen interaction [36], Amzn015 induced a rapid oxidative burst (0–12 h), marked by elevated O_2_^−^ and doubled SOD activity [37]. In the following phase (12–48 h), increased POD activity coupled with suppressed CAT maintained H_2_O_2_ as a defense signal, while preventing oxidative toxicity. By 48 h, MDA levels were significantly reduced, signaling effective mitigation of lipid peroxidation. This temporally coordinated modulation of ROS production and scavenging reinforces the notion that Amzn015 enhances host defense by amplifying early oxidative responses while minimizing later-stage cellular damage.

The apparent decline in CAT activity observed after Amzn015 treatment—contrary to reports that *B. amyloliquefaciens* AW3 enhances CAT in poplar [38]—likely reflects differences in pathogen pressure and ROS dynamics. First, Amzn015’s strong antagonism may reduce pathogen-induced oxidative stress, diminishing the need for CAT-mediated detoxification, akin to observations in poplar under milder infection [33]. Second, CAT’s role extends beyond mere ROS clearance—it also participates in redox signaling. Lower CAT activity may support sustained H_2_O_2_ signaling while avoiding toxicity, illustrating the complex regulatory balance within plant ROS metabolism. Moreover, Amzn015’s disease suppression is multifactorial—encompassing niche competition, antimicrobial production, immune activation, colonization, and interactions with the host microbiome, all converging to shape the plant’s defense response [39,40].

The present study aimed to determine whether *B. velezensis* Amzn015 mobilizes host immunity in *A. macrocephala* during *F. oxysporum* infection. Gene-expression analysis revealed that Amzn015 significantly upregulated key markers across both the SA-mediated SAR pathway (*NPR1*, *PR1*/*2*/*4*, *ICS*) and the JA/ET-associated ISR pathway (*JAR1*, *ERF1*, *ETR2*), surpassing expression levels in the *F. o* group. Traditionally, SAR and ISR are viewed as antagonistic with SA targeting biotrophs and JA/ET focusing on necrotrophs [41,42,43]. However, emerging evidence shows that beneficial microbes can trigger both pathways simultaneously. These findings suggest that Amzn015 orchestrates a coordinated activation of dual defense mechanisms, reinforcing the plant’s immune response beyond classical pathway separation. Several previous studies have demonstrated positive regulatory roles for WRKY transcription factors in plant defenses. For example, *LrWRKY2* from *Lilium regale* is induced by *F*. *oxysporum* infection and hormones such as SA and MeJA [44], and overexpression of *LrWRKY2* in transgenic tobacco significantly increases resistance. By contrast, in our *F.o* + Amzn015 treatment, *WRKY22* and *WRKY26* were downregulated compared to the *F.o* group, suggesting that Amzn015 may suppress these specific WRKYs under infection. This disparity may reflect a self–regulatory mechanism in plant immunity, where after initial activation of strong defense responses, certain WRKYs are repressed to balance defense costs, avoid overactivation, or due to feedback from hormone signaling.

This dual activation of SA and JA/ET signaling aligns with emerging evidence that some *Bacillus* spp., such as *Bacillus cereus* AR156, can co-activate SAR and ISR through an *NPR1*-dependent mechanism, thereby providing broad-spectrum protection against diverse pathogens [45]. Similar observations have been reported for *B. velezensis* BY6, which simultaneously induced both SA and JA/ET mediated pathways in *Pterocarya stenoptera*, resulting in a combined SAR + ISR defense effect [33]. These findings reinforce the idea that Amzn015 confers multifaceted immune priming by bridging classical pathway boundaries, enhancing host defense flexibility and robustness under fungal attack.

The data highlight that Amzn015 uniquely co-activates both SAR and ISR pathways in *A. macrocephala*—a rare feature among biocontrol agents. While many beneficial microbes trigger either the SA-dependent SAR or the JA/ET-dependent ISR, Amzn015 elicits sustained upregulation of both sets of marker genes (SA: *NPR1, PR1/2/4, ICS*; JA/ET: *JAR1, ERF1, ETR2*) under *F. oxysporum* challenge. Emerging studies show that such dual-pathway activation, often orchestrated via *NPR1*-dependent crosstalk, can produce additive or synergistic defense outcomes—supporting robust resistance to both biotrophic and necrotrophic threats [45]. Furthermore, microbial recognition via MAMP-triggered pathways, involving MAPK cascades, may underpin the simultaneous mobilization of SA and JA/ET signaling. Building on these insights, future work will dissect how *NPR1* mediates SA-JA/ET integration, map upstream events—such as PRR-MAMP recognition and MAPK activation—and explore hormonal interplay through hormone profiling, transcriptomics, and targeted functional studies. This mechanistic clarity will strengthen the theoretical framework for applying Amzn015 in sustainable, large-scale cultivation of *A. macrocephala.*

## 5. Conclusions

This study is the first to show that endophytic *B. velezensis* Amzn015, isolated from *A. macrocephala*, combines potent antifungal activity in vitro with effective root rot suppression and growth recovery in infected seedlings. Mechanistically, Amzn015 dynamically modulates ROS metabolism, co-activates SA–JA/ET mediated immune pathways, and boosts phenylpropanoid biosynthesis—resulting in an integrated SAR and ISR defense. These dual benefits in disease resistance and plant vigor, coupled with its safety profile, position Amzn015 as a promising green biocontrol agent, providing a novel microbial resource and mechanistic insights.

## Data Availability

The original data presented in this study are included in this article, and the 16S rRNA gene sequence of the strain are openly available in the National Center for Biotechnology Information (NCBI) database under accession number PV972215. The whole-genome sequence of the strain are openly available in the data NCBI database under accession number PRJNA1294532.

## Supplementary Materials

The following supporting information can be downloaded at: https://www.mdpi.com/article/10.3390/microorganisms13102300/s1, Figure S1: Phylogenetic tree of three fungal strains constructed based on ITS sequences. Table S1: The primers in this study; Table S2: Antagonistic activities of 10 endophytic bacteria against *Fusarium oxysporum* in dual culture.
