# Supplementary material for: Dual Benefits of Endophytic Bacillus velezensis Amzn015: Growth Promotion and Root Rot Control in Atractylodes macrocephala
Source: Microorganisms. 2025 Oct 3;13(10):2300. doi: 10.3390/microorganisms13102300 (PMC12566287; doi:10.3390/microorganisms13102300)
Supplement: Supplementary file 1 [file microorganisms-13-02300-s001.zip › Supplementary Figure S1.pdf]

Supporting Information

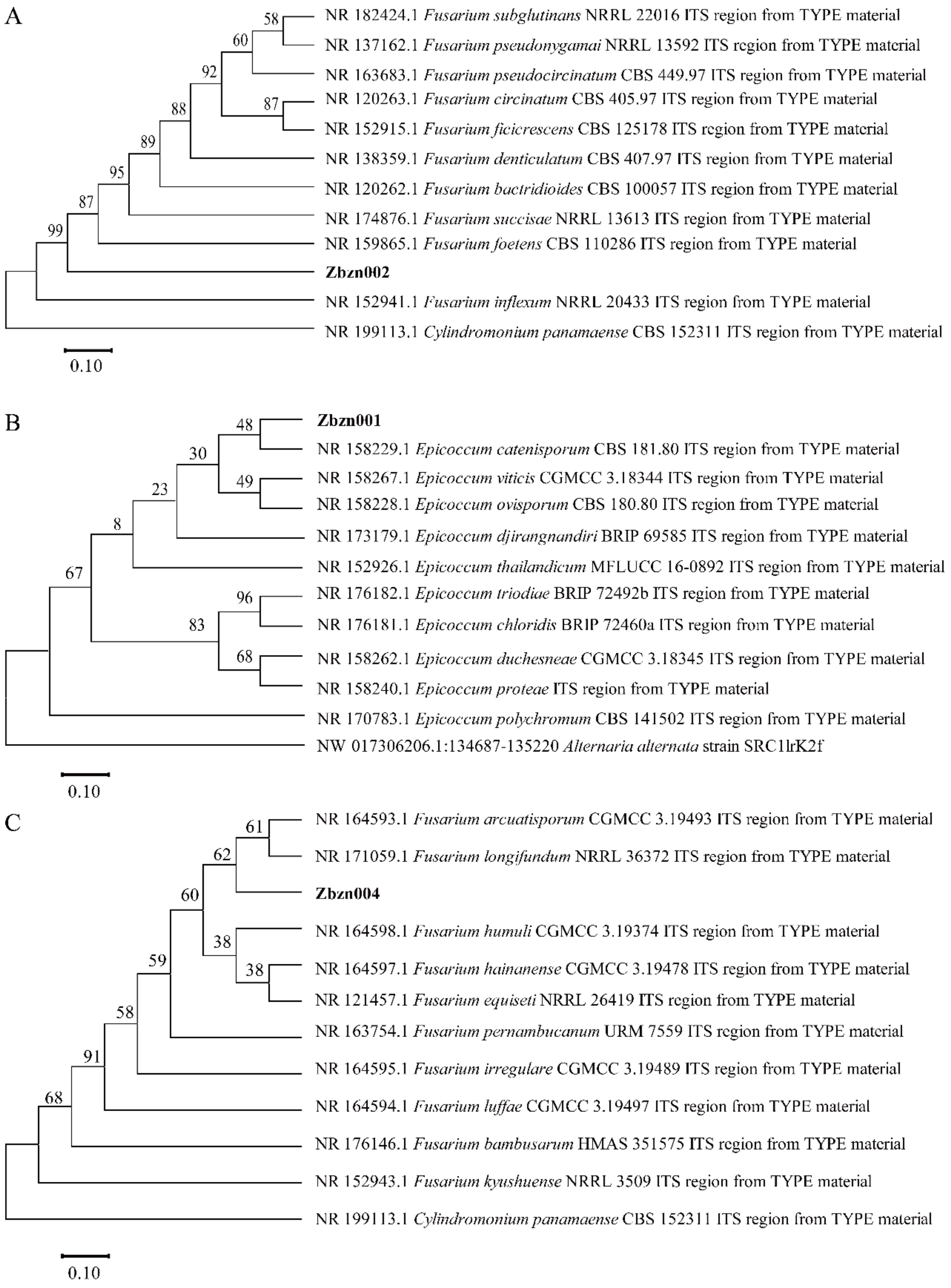

Figure S1. Phylogenetic tree of three fungal strains constructed based on ITS sequences. The tree was generated using the maximum likelihood (ML) method in MEGA 11, with sequence alignment performed using ClustalW. Reference sequences were retrieved from the NCBI GenBank database to evaluate the phylogenetic positions of strains Zbzn002, Zbzn001, and Zbzn004. (A) Zbzn002 clustered within the *Fusarium* genus, (B) Zbzn001 was

identified as belonging to the *Epicoccum* genus, and (C) Zbzn004 also clustered with the *Fusarium* genus, supporting the molecular identification of all three isolates.
